# A Novel Flexible Room Temperature Ethanol Gas Sensor Based on SnO_2_ Doped Poly-Diallyldimethylammonium Chloride

**DOI:** 10.3390/s130404378

**Published:** 2013-04-02

**Authors:** Shuang Zhan, Dongmei Li, Shengfa Liang, Xin Chen, Xia Li

**Affiliations:** 1 Key Laboratory of Nano-Fabrication and Novel Devices Integrated Technology, Institute of Microelectronics, Chinese Academy of Science, Beijing 100029, China; E-Mails: zhanshuang@ime.ac.cn (S.Z.); liangshengfa@ime.ac.cn (S.L.); chenxin@ime.ac.cn (X.C.); 2 College of Materials Science and Technology, Qing Dao University of Science &Technology, Qingdao 266042, China

**Keywords:** flexible, ethanol gas sensor, SnO_2_, PDDAC, room temperature

## Abstract

A novel flexible room temperature ethanol gas sensor was fabricated and demonstrated in this paper. The polyimide (PI) substrate-based sensor was formed by depositing a mixture of SnO_2_ nanopowder and poly-diallyldimethylammonium chloride (PDDAC) on as-patterned interdigitated electrodes. PDDAC acted both as the binder, promoting the adhesion between SnO_2_ and the flexible PI substrate, and the dopant. We found that the response of SnO_2_-PDDAC sensor is significantly higher than that of SnO_2_ alone, indicating that the doping with PDDAC effectively improved the sensor performance. The SnO_2_-PDDAC sensor has a detection limit of 10 ppm at room temperature and shows good selectivity to ethanol, making it very suitable for monitoring drunken driving. The microstructures of the samples were examined by scanning electron microscopy (SEM), X-ray diffraction (XRD), transmission electron microscope (TEM) and Fourier transform infrared spectra (FT-IR), and the sensing mechanism is also discussed in detail.

## 1. Introduction

Ethanol gas sensors can be applied in many fields, such as the control of fermentation processes [1], safety testing of food packaging, and can also be fixed on vehicle steering wheels to monitor drunken driving [2,3]. Recently, plastic substrate-based ethanol sensors have attracted considerable attention, owing to their attractive characteristics including flexibility, lightness, shock resistance, and softness. However, most plastics will deform or melt at temperatures of only 100–200 °C [4], causing severe limitations on sensor application as many gas sensors are required to operate at high temperature (>200 °C), so we have focused our attention on the development of flexible sensors for the detection of ethanol at room temperature, which not only avoids the need for heaters on the substrates, but also makes the assembly of the sensors much simpler, cheaper and more portable [5].

Metal oxides like SnO_2_, WO_3_, ZnO, γ-Fe_2_O_3_, have been extensively studied in the gas sensing area [6–9]. SnO_2_ is frequently used to detect ethanol due to its many advantages such as simple manufacturing technique, low cost, and rapid response and recovery time [2], but generally it requires a high working temperature beyond 300 °C. There are also several organic semiconductors, such as polythiophene, polypyrrole, polyaniline [10–14], that have been used for detecting gases, however, poor selectivity is the most serious problem for inorganic and organic conducting polymer sensing materials. To meet the need of analyzing gas mixtures, and to overcome the poor selectivity and high cost problem of popular sensors, organic-inorganic hybrid composite sensors are being intensively investigated [15–18]. They can complement the disadvantages of pure inorganic and organic materials. It is also observed that hybrid materials have smaller grain size and better gas-sensing stability in air [19,20]. Geng [21] reported that the polyaniline/SnO_2_ hybrids exhibited good sensitivity to volatile organic compounds. Ram *et al*. [17] synthesized poly(ethylenedioxythiophene) (PEDOT)/SnO_2_ composite thin films, and studied their gas sensitivity to NO_2_. These hybrid materials-based gas sensors exhibited much higher sensitivity than that of the pure inorganic and organic materials-based gas sensors.

However, the adherence between the sensing layer and the substrate is then of outmost importance. The ceramic substrate-based sensors usually need to use an inorganic binder to promote the adhesion between the components [22]. For flexible organic substrates, elevated temperatures should be avoided and generally polymeric sensitive materials with intrinsic binding properties are necessary.

PDDAC is frequently used as a binder in the electrodeposition of iron oxide films, which enables the formation of thick metal oxide films, preventing cracking of the film and increasing the adhesion between the sensitive film and the substrates [23]. It can also be used to adjust the electrostatic force between flexible fibers and inorganic filler particles, thus facilitating the retention of fillers [24]. Moreover, considering that PDDAC is a charged polyelectrolyte, the electrostatic interaction between PDDAC and metal oxide may modify the sensing properties of the mental oxide at room temperature.

In this paper, we have investigated a novel flexible ethanol sensor based on SnO_2_ doped polydiallyldimethylammonium chloride (PDDAC), in which PDDAC acted as both the binder and the dopant. The sensor has a detection limit of 10 ppm and shows good selectivity to ethanol. Furthermore, the sensing mechanism is also discussed in detail.

## 2. Experimental

### 2.1. Materials Preparation

SnO_2_ (AR, purity ≥ 99%) and PDDAC (M.W. = 100,000−200,000 g/mol) were purchased from Tianjin Wind Ship Co. Inc., Tianjin, China. They were used as received without any treatment. All de-ionized water (DIW) used had a resistance above 18 MΩ/cm. The PI substrate (Upilex-125S, UBE, Japan) was washed with acetone, ethanol, and DIW, respectively.

### 2.2. Fabrication of Gas Sensor

Interdigitated gold electrodes were formed on the flexible PI substrate (10 mm × 11 mm) by E-beam evaporation of a thin (5 nm) layer of Cr, serving as the adhesion layer and then a 50 nm Au layer. The electrodes have four pairs of interdigital fingers, each of 4,950 μm length and 50 μm width and the gap between the electrodes is also 50 μm. The solution of PDDAC was formed by dissolving 2.5 g PDDAC in 25 mL DIW at 298 K. Then 2.0 g SnO_2_ nanopowder was added to the as-prepared PDDAC solution and the mixture was ultrasonicated for 1 h to give a homogenous saturated solution at 298 K. Ten μL of the saturated solution were coated onto the interdigitated electrodes by drop casting and dried in air at 353 K for 15 min. For the SnO_2_ sensor fabrication, 2.0 g SnO_2_ was dissolved in 25 mL DIW and the mixture was ultrasonicated for 1h to give a homogenous saturated solution at 298 K, after which 10 μL of the prepared SnO_2_ solution was also coated onto the interdigitated electrodes by drop coating and then dried in air at 353 K for 15 min. All experiments were conducted at ambient conditions with a temperature of 24.5 ± 0.5 °C and relative humidity of 45 ± 5%.

### 2.3. Characterization and Gas Sensing Measurement System

**Figure 1.**
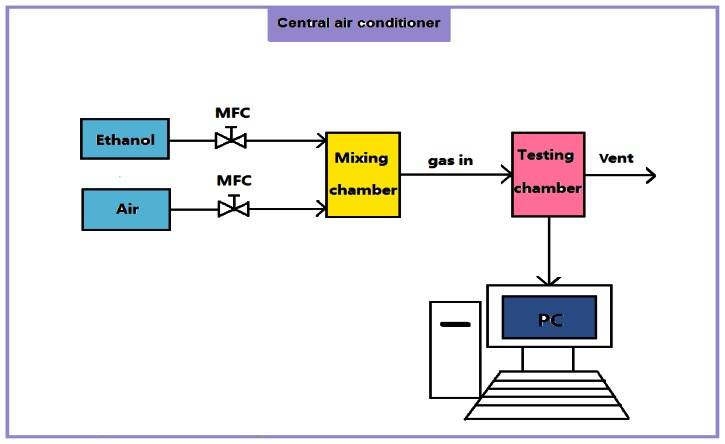
Gas sensing measurement system.

The morphologies of SnO_2_-PDDAC and SnO_2_ films were characterized by a SEM (XL30S-FEG, FEI, The Netherlands) equipped with an EDX detector (EDAX Instruments, USA). The mean grain size was analyzed by X-ray diffraction (Bruker D8 Focus, Germany). The detailed characterization of the SnO_2_-PDDAC sample was carried out by TEM (JEM-1011, JEOL, Japan). FT-IR (Excalibur 3100, Varian, USA) was used to characterize the components of each film. The gas sensing tests were performed by the gas sensing measurement system (NSSRL-811, Kena Smart Instruments, Wuhan, China), as shown in Figure 1. As the figure shows, two mass flow controllers (MFCs) were used to control the flow rate of synthetic air (dry air), the carrier gas, and ethanol, the target gas, respectively. The gases were purchased from the Beijing Tai Long Electron Technology Co. Ltd., Beijing, China. The carrier gas and target gas were mixed in the mixing chamber and then were introduced to the testing chamber. A PC was connected to the testing circuit to monitor and record the resistance of the sensor. The temperature and humidity of the testing room were controlled by a central air conditioner. The gas sensing measurement was conducted by exposing the sensor in ethanol for 10 min and air for 10 min, respectively. The flow rate of the gas is 500 mL/min, and the volume of the chamber is 275 mL.

## 3. Result and Discussion

### 3.1. Ethanol Sensing Tests

The sensor response (*S*) was defined as:

(1) $$S=({\text{R}}_{\;\text{gas}}-{\text{R}}_{\;\text{air}})\times 100/{\text{R}}_{\;\text{air}}=\Delta \text{R}\times 100/{\text{R}}_{\;\text{air}}$$

**Figure 2.**
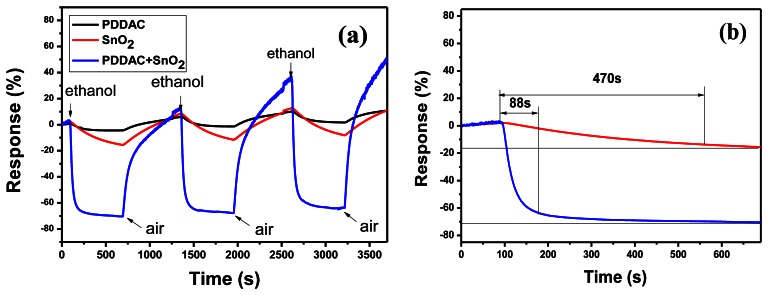
(**a**) Response of the SnO_2_–PDDAC and SnO_2_ sensor to 150 ppm ethanol at room temperature; (**b**) The response time of the SnO_2_-PDDAC and SnO_2_ sensors to 150 ppm ethanol.

In Equation (1), R_gas_ and R_air_ are the electrical resistance when exposed to ethanol and air, respectively. The gas sensing properties of SnO_2_-PDDAC and SnO_2_ sensors at room temperature were both tested. Figure 2(a) shows the typical response of SnO_2_-PDDAC, SnO_2_ and PDDAC sensors to 150 ppm ethanol. The response of the SnO_2_ and PDDAC sensors to 150 ppm ethanol was used as the reference. The response time is defined as the time of the sensor needs to reach 90% of the equilibrium value after the injection of the test gas.

As Figure 2(a) shows, the SnO_2_ and PDDAC sensors show a reversible response to ethanol, but the response is much lower than that of SnO_2_-PDDAC sensor. From Figure 2(b) we can see that the response of SnO_2_-PDDAC and SnO_2_ sensor to 150 ppm ethanol at room temperature is about 71.6% and 15.5%, and the response time was about 88 s and greater than 470 s, respectively. The SnO_2_-PDDAC sensor has higher response and shorter response time, so we chose SnO_2_-PDDAC sensor as the sample for the further ethanol sensing tests.

**Figure 3.**
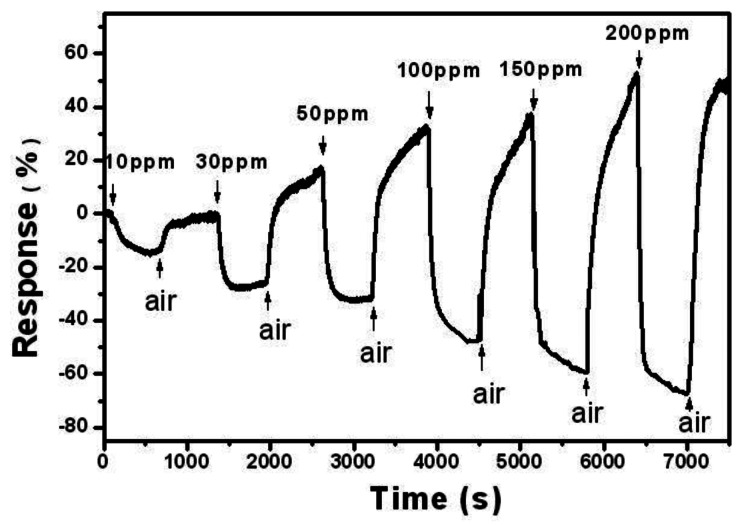
Response of SnO_2_-PDDAC sensor to ethanol of different concentrations.

Figure 3 shows the response of SnO_2_-PDDAC sensor to different concentrations of ethanol. The detection limit of our sensor is 10 ppm at room temperature, but noticeable drift of base resistance was observed after each response-recovery cycle, and similar phenomenon can be observed in Figure 2(a), which may be related to the incomplete desorption of gas on the sensor at room temperature. Such incomplete gas adsorption prolongs the recovery time [25], and the drift amplitude is observed to increases continuously with increasing the gas concentration. Further research is required to understand the interrelation between the gas concentration and the gas adsorption.

**Figure 4.**
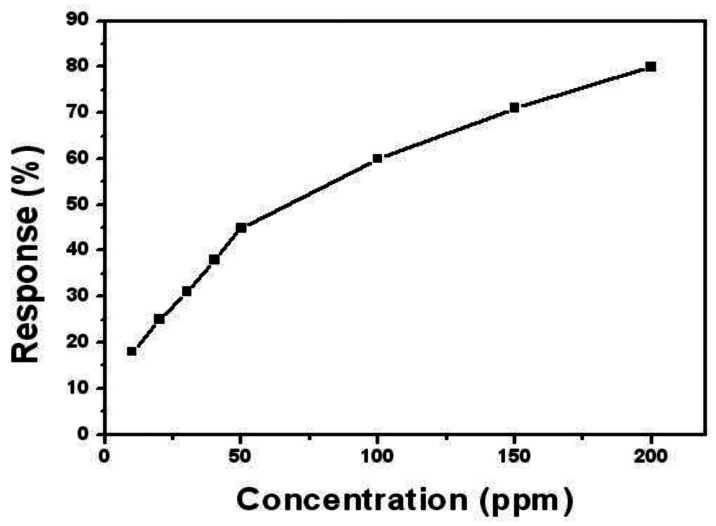
The normalized response of the SnO_2_-PDDAC sensor to ethanol of different concentrations.

Figure 4 shows the relationship between the response and the concentrations of ethanol. The sensor shows linear response to ethanol ranging from 10 to 50 ppm and 50 to 200 ppm, respectively. This can be explained reasonably by the adsorption of ethanol molecular on the surface of the SnO_2_-PDDAC film. As for low concentrations of ethanol (10–50 ppm), there are enough active sites for the ethanol molecules to be adsorbed. However, as the concentration of ethanol increases (50–200 ppm), there are not enough active sites for the ethanol adsorption, causing a decrease of the response curve's slope.

### 3.2. Characterization of Sensitive Films

**Figure 5.**
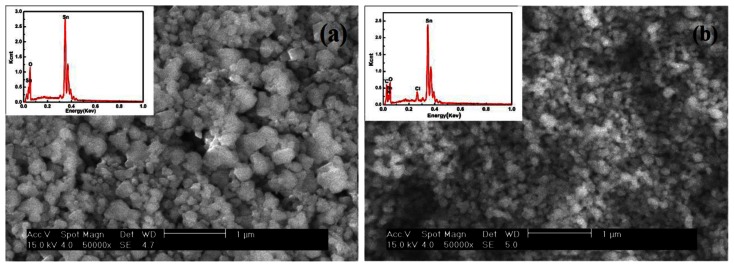
Surface morphology of the sensing film at the same magnification, (**a**) SnO_2_; (**b**) SnO_2_-PDDAC. The inset reports the EDX spectrum collected from the surface.

Figure 5 shows the SEM images and EDX spectra of SnO_2_ (Figure 5(a)) and SnO_2_-PDDAC (Figure 5(b)). The SnO_2_ nano-particles in the SnO_2_-PDDAC film are more uniformly dispersed than in SnO_2_ film. This may be attributed to the interaction between SnO_2_ and PDDAC. PDDAC is a polyelectrolyte, so the electrostatic interaction between the SnO_2_ and PDDAC, together with the steric effect of the polymer [26] are likely to prevent the metal oxide from aggregating and make the hybrid materials have uniform grain size.

The components of the films are characterized by EDX (see the inset in Figure 5). The presence of SnO_2_ particles is indicated by O and Sn, and PDDAC is indicated by C, N and Cl.

**Figure 6.**
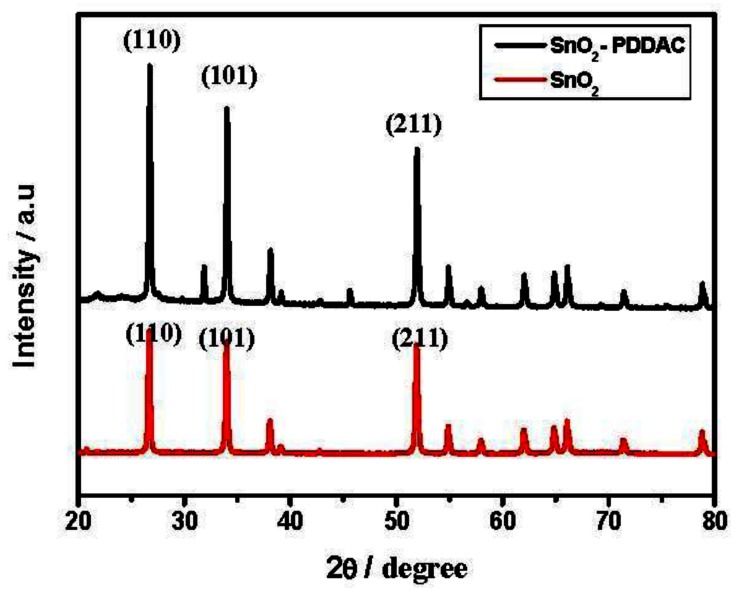
XRD patterns of SnO_2_ and SnO_2_-PDDAC film.

X-ray diffraction patterns of SnO_2_, SnO_2_-PDDAC are shown in Figure 6. The mean grain size was calculated by the Debye-Scherrer formula:

(2) d=0.9λ/Bcosθ

in which d is the crystalline diameter, λ is X-ray wavelength, B is line broadening measured at half height and θ is Bragg angle, giving a mean grain size of 37 ± 2 nm for SnO_2_ and 36 ± 2 nm for SnO_2_-PDDAC samples, according to the crystalline plane (110). The mean grain size of the two samples is similar, but the grains agglomerate more severely in the SnO_2_ film than in the SnO_2_-PDDAC film, as shown in the SEM images.

**Figure 7.**
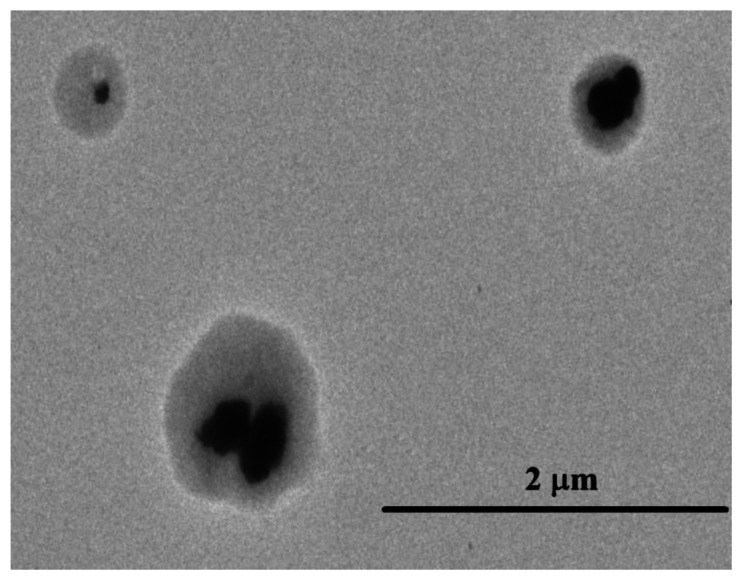
TEM image of SnO_2_-PDDAC hybrid material.

To further confirm the morphology of the SnO_2_-PDDAC sample, TEM investigation was performed. Figure 7 shows a TEM image of the SnO_2_-PDDAC hybrid material. It clear from the figure that SnO_2_ is observed as black dots capsulated in PDDAC.

**Figure 8.**
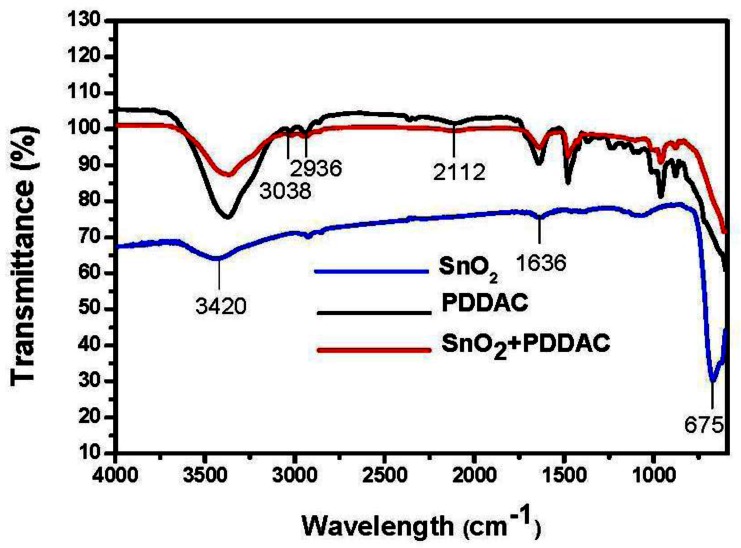
FT-IR spectra of SnO_2_, PDDAC and SnO_2_-PDDAC film.

The composition of the sensitive films was investigated by FT-IR. As shown in Figure 8, due to the fact the SnO_2_ is capsulated in PDDAC, the characteristic peaks of SnO_2_ are not evident in the spectrum of the SnO_2_-PDDAC film. The broad bands around 3,420 cm^−1^ and the band centered at 1,636 cm^−1^ found in the materials are assigned to O-H stretching, which is caused by the vibrations of adsorbed water molecules. Due to the hygroscopicity of PDDAC, the band of PDDAC at 3,420 cm^−1^ observed is higher than that of SnO_2_-PDDAC and tin oxide film. The stretching vibration of N-C bond is centered at 2,112 cm^−1^, and the band centered at 675 cm^−1^ is attributed to the framework vibrations of tin oxide [27]. In addition to the bands ascribed to SnO_2_ species, bands at 2,936 cm^−1^ and 3,038 cm^−1^ observed in the PDDAC and SnO_2_-PDDAC spectra are the C-H stretching vibration adsorption.

**Figure 9.**
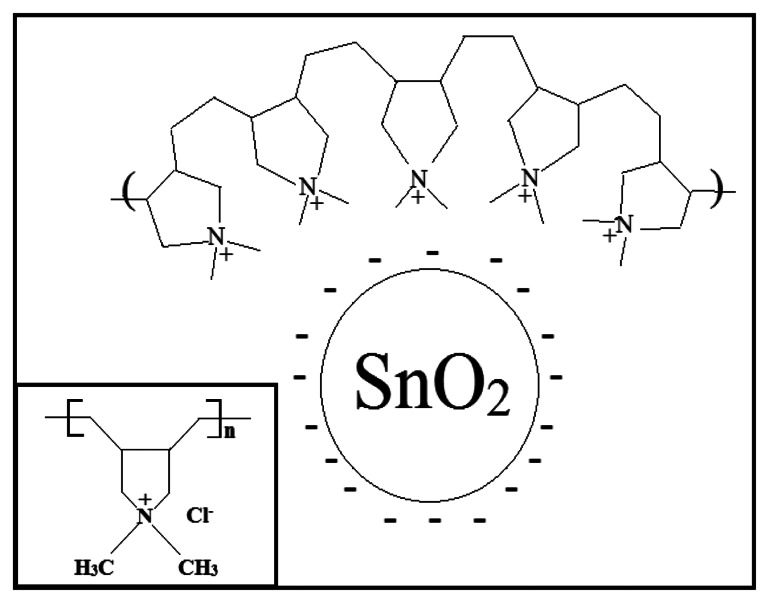
The electrostatic interaction between SnO_2_ and PDDAC. The inset is the chemical structure of PDDAC.

In order to understand the sensing mechanism of SnO_2_-PDDAC sensor to ethanol, the following facts should be taken into consideration: (i) particles are likely to acquire surface charges when they are in contact with an aqueous solution [28]; the particles of metal oxide in water become hydroxylated, forming M-OH groups on the surface, which can be ionized to give positive or negative charges, depending on the metal oxide point of zero charge (pzc). Values of pzc for SnO_2_ are in the 3.5–4.5 range, therefore when dissolved in neutral water, the surface of SnO_2_ is negatively charged; (ii) PDDAC is a polyelectrolyte with a positive charge located on the quaternary ammonium group (see the inset in Figure 9). Indeed, such an ionizable group dissociates fully in water solutions, releasing counterions (*i.e.*, Cl^−^), and leaving the positively charged polymer segments [5].

As Figure 2 shows, SnO_2_-PDDAC sensor is more sensitive to ethanol than the SnO_2_ and PDDAC sensors, and the response and recovery are also much faster. This may be attributed to the effective interaction between SnO_2_ and PDDAC, as shown in Figure 9. The details can be explained as follows: when dispersed in water solution, the chains expand as the positive charges on the polyelectrolyte chain repel each other. When mixed with SnO_2_, SnO_2_ grains will embed into the chains of PDDAC because of the electrostatic interaction between the positive charges on the polyelectrolyte PDDAC chain and the negative charge on the surface of the SnO_2_ nanoparticles. The interaction between the oxide and polymer plays an important part in the sensing mechanism of these composite sensors according to previous reports [29,30]. SnO_2_ nanoparticles disperse more uniformly due to the electrostatic interaction between PDDAC and SnO_2_, which increases the surface area of polymer when exposed to ethanol and creates a porous network and thus leads to the increase of gas sensing efficiency [5,29–33]. On the other hand, SnO_2_ and PDDAC can form heterojunctions. When the p-n junction between SnO_2_ and PDDAC is formed, the electrons from SnO_2_ will diffuse across the junction and recombine with holes in PDDAC; similarly, some of the holes in PDDAC will diffuse across the junction and recombine with free electrons in SnO_2_. This will form a depletion layer. The p-type PDDAC thus acquires a slight negative charge and the n-type SnO_2_ acquires a slight positive charge. The p-n junction formed between them may cause a lower activation energy and enthalpy of physisorption for vapors with good electron- donating characteristics [34], which makes it easier for the adsorption of ethanol molecules, leading to the increase of sensor response.

### 3.3. Stability and Selectivity of the Sensor

**Figure 10.**
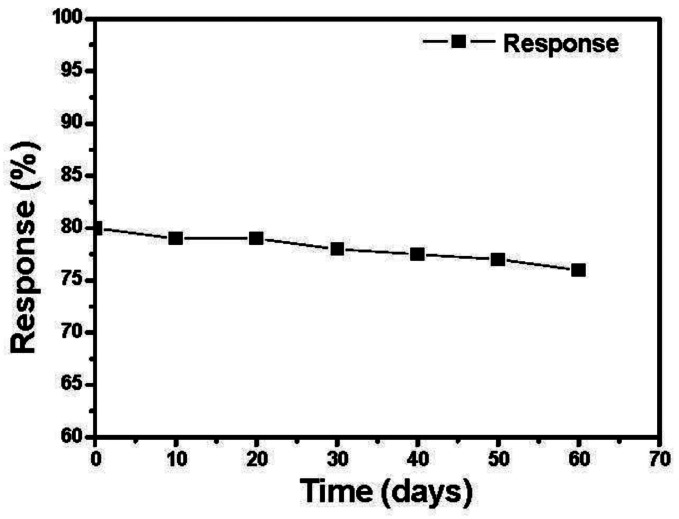
The response of the SnO_2_-PDDAC sensor as a function of time (days).

We tested the response of the sensor every ten days after their fabrication. As shown in Figure 10 the sensors have a nearly constant response to 200 ppm ethanol during two months.

**Figure 11.**
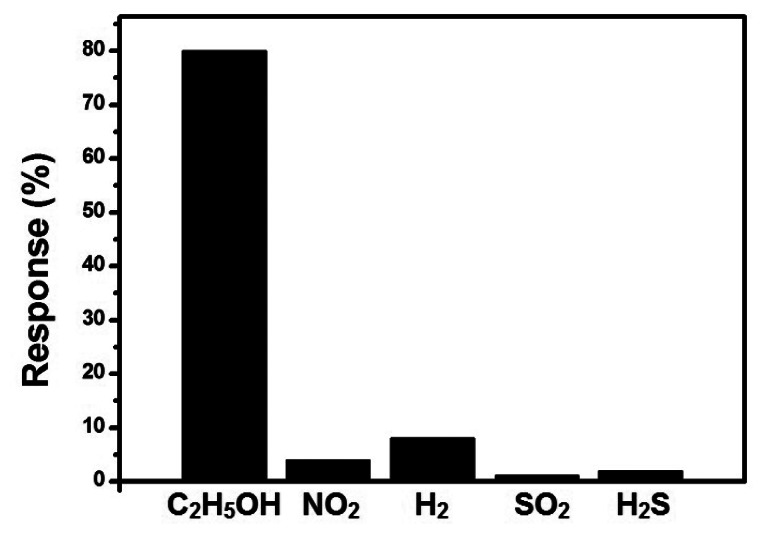
Response of the SnO_2_-PDDAC sensor to 200 ppm C_2_H_5_OH, NO_2_, H_2_, SO_2_, and H_2_S.

As selectivity is also very important for a gas sensor, we tested the selectivity of the sensor by exposing the sensor to different target gases of the same concentration, including both oxidizing and reducing gases (C_2_H_5_OH, NO_2_, H_2_, SO_2_, and H_2_S), as shown in Figure 11. From the figure, we can see that our sensor has good selectivity to C_2_H_5_OH as the response to other gases of the same concentration is significantly smaller than that of C_2_H_5_OH.

## 4. Conclusions

In this paper we have demonstrated a room temperature, low cost and flexible ethanol sensor using SnO_2_-PDDAC as sensitive film. We found that PDDAC not only served as the binder, but also contributed to the improvement of the sensor's performance. The detection limit of SnO_2_-PDDAC sensor is 10 ppm at room temperature, and it has good durability over at least two months. The sensor also shows good selectivity to ethanol. These favorable gas sensing features make the proposed SnO_2_-PDDAC sensor a potential candidate for monitoring ethanol at room temperature.
